# A Christ's Mass in Hospital

**Published:** 1887-12-24

**Authors:** Jessie M. E. Saxby


### A CHRIST'S MASS IN HOSPITAL.

Christ of the wounded hands which heal,
Be in our house of pain;
That aching forms Thy touch may feel,
And rest again.

Christ of the cross and crown of thorn
Be near us where we lie,
To teach each heart by suffering torn
How heroes die.

Christ of the conquest over ill,
Christ of immortal life,
Say to our anguished souls, "Be still,"
So calm their strife.

Christ who alone canst heal and save,
Come, and our fears shall cease.
Oh, make us in our weakness brave,
And give us peace.

Jessie M. E. Saxby.

